# miR-205-5p Modulates High Glucose-Induced VEGFA Levels in Diabetic Mice and ARPE-19 Cells

**DOI:** 10.3390/antiox14020218

**Published:** 2025-02-14

**Authors:** María Ybarra, Miriam Martínez-Santos, Maria Oltra, María Muriach, Maria E. Pires, Chiara Ceresoni, Javier Sancho-Pelluz, Jorge M. Barcia

**Affiliations:** 1 Escuela de Doctorado Universidad Católica de Valencia San Vicente Mártir, 46001 Valencia, Spain; maria.ybarra@ucv.es (M.Y.); miriam.msantos@ucv.es (M.M.-S.); me.dossantos@ucv.es (M.E.P.); chiara.ceresoni@ucv.es (C.C.); jm.barcia@ucv.es (J.M.B.); 2 Departamento de Anatomía y Fisiología, Facultad de Medicina y Ciencias de la Salud, Universidad Católica de Valencia San Vicente Mártir, 46001 Valencia, Spain; maria.oltra@ucv.es; 3 Centro de Investigación Traslacional San Alberto Magno, Universidad Católica de Valencia San Vicente Mártir, 46001 Valencia, Spain; 4 Departamento de Medicina, Facultad de Ciencias de la Salud, Universidad Jaime I, Avda. Vicent Sos Baynat, 12006 Castellón de la Plana, Spain; muriach@med.uji.es

**Keywords:** microRNA, angiogenesis, retinal pigment epithelium, diabetes, oxidative stress

## Abstract

High glucose levels may cause vascular alterations in patients with diabetes, which can lead to complications such as diabetic retinopathy—an abnormal growth of retinal blood vessels. The micro-RNA miR-205-5p is known to regulate angiogenesis by modulating the expression of the vascular endothelial growth factor (VEGFA) in different systems. This study investigates the role of miR-205-5p in controlling VEGFA expression both in vitro and in the eye under hyperglycemic conditions. An alloxan-induced diabetic mouse model and retinal pigment epithelium human cell line (ARPE-19) were exposed to high glucose and treated with an ectopic miR-205-5p mimic. VEGFA mRNA and protein levels were assessed using qRT-PCR, Western blot, and immunocytochemistry. Additionally, human umbilical vein endothelial cells (HUVECs) were employed to evaluate angiogenesis. Our results show that high glucose significantly reduced miR-205-5p levels while upregulating VEGFA expression in both ARPE-19 cells and diabetic mice. The ectopic administration of miR-205-5p (via transfection or intravitreal injection) restored VEGFA levels and inhibited angiogenesis in HUVEC cultures. Based on these preliminary data, we suggest a potential therapeutic strategy against VEGFA involving miR-205-5p in proliferative eye-related vascular disorders.

## 1. Introduction

Diabetes mellitus is a chronic disease characterized by systemic complications closely tied to vascular alterations, including nephropathy, cardiac failure, and blindness [1]. Among the affected tissues, the retinal pigment epithelium (RPE) plays a critical role. This highly specialized monolayer of cells forms a connection between the vascular choroid and the photoreceptor cell layer, maintaining the blood–retinal barrier (BRB) [2]. The BRB facilitates communication between the choroid and the retina, ensuring the appropriate exchange of nutrients and metabolic waste [3]. However, hyperglycemia in diabetes disrupts these functions, leading to microvascular damage, even in the early stages of the disease [4].

Hyperglycemia-induced vascular alterations are central to the progression of diabetic retinopathy (DR). Early molecular changes include oxidative stress, inflammation, and pericyte loss, which contribute to increased vascular permeability and neovascularization [5]. These alterations are closely tied to the upregulation of vascular endothelial growth factor A (VEGFA) [6]. Under physiological conditions, the RPE secretes VEGFA to maintain choroidal endothelial survival and fenestration. However, high glucose conditions lead to overproduction, exacerbating vascular permeability and promoting leaky blood vessels [7].

MicroRNAs (miRNAs) are highly conserved non-coding RNAs discovered in the early 1990s [8]. These molecules regulate gene expression by degrading or inhibiting mRNA transcription targets [9]. Advances in miRNA research have shifted from early identification to therapeutic applications within the last decade [10]. miRNAs hold potential for treating proliferative vascular diseases like DR, particularly in cases where VEGFA antibody therapies fail to produce a response [11]. Among these, miR-205-5p, first identified in the mammalian eye in 2006 [12], has garnered attention due to its multifaceted roles in anti-proliferative pathways [11,12,13], maintenance of epithelial characteristics [14], and regulation of oxidative stress and angiogenesis [12,15].

The 2024 Nobel Prize in Physiology and Medicine awarded to Victor Ambros and Gary Ruvkun underscores the significance of miRNA research, recognizing their transformative role in understanding and targeting diseases at the molecular level [16]. miR-205-5p, in particular, is highly conserved across species and is expressed predominantly in epithelial tissues. Recent studies suggest that hyperglycemia can induce epigenetic modifications affecting miR-205-5p transcription [17], which in turn disrupts its regulatory functions on VEGFA [18]. Interestingly, miR-205-5p interacts with VEGFA through a well-conserved single binding site located in the 3′UTR, underscoring its potential as a precise therapeutic tool [19].

This study investigates the therapeutic potential of miR-205-5p in modulating VEGFA expression under high glucose conditions. By leveraging both in vivo and in vitro models, we aim to provide foundational insights into the molecular mechanisms underlying its anti-angiogenic efficacy. These findings contribute to the broader objective of identifying miR-205-5p as a novel therapeutic candidate for proliferative vascular diseases such as DR.

## 2. Materials and Methods

### 2.1. Cell Culture 

The retinal pigment epithelium human cell line (ARPE-19) was obtained from the American Type Culture Collection (ATCC, Manassas, VA, USA). ARPE-19 cells were cultured for 5 days in Dulbecco’s modified Eagle DMEM/F12 (Invitrogen, Carlsbad, CA, USA) as previously described [18]. Cells were used from 11 to 30 passages. Cells were cultured to 80–90% confluence at a starting density of 1 × 10^6^ cells/cm^2^ in different plates, depending on the technique. ARPE-19 cells were incubated with high glucose (HG group, 35 mmol/D glucose) (Sigma-Aldrich, St. Louis, MO, USA) or control glucose (CG 5.5 mmol/L glucose) (Sigma-Aldrich, St. Louis, MO, USA) with 19.5 mmol/L mannitol (Sigma-Aldrich, St. Louis, MO, USA) to exclude any potential bias due to osmotic effect. As high glucose exposure results in an oxidative challenge for cells and tissues, we used N-Acetylcysteine (NAC) as an antioxidant to check the role of oxidative stress in glucose-induced angiogenesis. For this reason, NAC was used at a concentration of 4 mM (Sigma-Aldrich, St. Louis, MO, USA). A 1% fetal bovine serum (FBS; Thermo Fisher Scientific, Waltham, MA, USA), penicillin/streptomycin, and amphotericin 1% were supplied under all conditions. Human umbilical vein endothelial cells (HUVECs) were isolated from umbilical veins as previously described [18]. HUVECs were grown in endothelial cell media (Innoprot, Derio, Spain) supplemented with 20% FBS, penicillin/streptomycin, and amphotericin 1% at 37 °C and 5% CO_2_ for angiogenesis and migration experiments.

### 2.2. Cell Culture-Conditioned Media (CCCM) 

Cell culture-conditioned media were collected from ARPE-19 after 5 days of treatment for angiogenesis and scratch wound healing assays with HUVEC cells. CCCM included the control; control + mimic (miR-205-5p); 35 mM glucose; and 35 mM glucose + mimic (miR-205-5p).

### 2.3. Scratch Wound Healing Assay

HUVECs were seeded at 5 × 10^4^ cells/cm^2^ density in a 24-well plate during 48 h at 37 °C and 5% CO_2_. A wound was mechanically produced by scratching a cell monolayer with a sterile 200-µL pipette tip across the center of the well with cells attaining 90% confluence as previously described [19]. After 48 h, the CCCM from ARPE-19 were added (see Materials and Methods Section 2.2). Cells were then washed with PBS each time to avoid cell debris. Images were taken at 0, 4, 8, and 24 h using an Olympus CKX41 inverted microscope (Olympus, Tokyo, Japan) and recorded using an Olympus DP74 digital camera (Olympus, Tokyo, Japan). The gap distance was analyzed by ImageJ (Version 1.54m), per the following protocol.

### 2.4. Cell Viability

Cell viability was measured using 3′-[1-phenylaminocarbonyl-3,4-tetrazolium]-bis(4-methoxy-6-nitro) benzene sulfonic acid hydrate (XTT; Cell Viability CyQUANT^TM^; (Thermo Fisher Scientific, Waltham, MA, USA). 

### 2.5. Mimic Transfection ARPE-19

At 60–80% confluence, ARPE-19 cells were transfected with an miR-205-5p mirVana^®^ miRNA mimic (Thermo Fisher Scientific, Waltham, MA, USA) 30 pmol (10 µM) using a Lipofectamine 2000 RNAiMAX reagent (Thermo Fisher Scientific, USA) diluted in opti-MEM^®^ Medium (Thermo Fisher Scientific, Waltham, MA, USA). After 48 hours of transfection, the conditioned cell culture media and cell pellet were collected and stored at −80 °C for further assays.

### 2.6. Animals and Diabetic Model

All animal experiments were performed strictly following the ARVO statements for the use of animals in ophthalmic and visual research and were approved by the Ethics Committee for Research (Universidad Jaime I Castellón, ref. 21/12/2022). SWISS male mice were obtained from Janvier Laboratories (Le Genest-Saint-Isle, France). To induce diabetes, 200 mg/kg of alloxan monohydrate (Sigma Aldrich, St. Louis, MO, USA) was injected subcutaneously using a 25-gauge needle. 48 h after alloxan injection, mice with blood glucose levels ≥200 mg/mL were considered for a diabetic group. Animal welfare was daily checked by the lab veterinary, and blood glucose levels were measured once a week, as indicated below. VetPen insulin (MSD Animal Health, Rahway, NJ, USA) was used for those animals with signs of polyuria and polydipsia. Ninety-three mice were randomly divided in two groups (control or diabetic). After alloxan injections, some animals died or did not respond to alloxan (n = 10). Control (n = 40) and diabetic (n = 40) groups were randomly divided into 2 subgroups/each: control, control + mimic (miR-205-5p), diabetic, and diabetic + mimic (miR-205-5p) n = 20 animals per group were used. An independent experimental group (n = 3) was used to test the effect of Invivofectamine as a vehicle.

### 2.7. Intravitreal Injection

An miR-205-5p mirVana^®^ miRNA mimic (ThermoFisher, Waltham, MA, USA) was complexed with Invivofectamine 3.0 (ThermoFisher, Waltham, MA, USA), giving a final concentration of 2 mg/mL. Control and diabetic mice were randomly divided into two groups (injected with mimic or control) (n = 20/group). To avoid unnecessary suffering, each mouse received one intravitreal injection into the right eye. The left eye was used as a naïve control. To assess the effect of the vehicle, 2 mg/mL of Invivofectamine 3.0 (ThermoFisher, Waltham, MA, USA) was intravitreally injected into the right eye in an independent group n = 3 (vehicle). Four weeks after diabetes induction, the animals received one intravitreal injection under a 1:1 cocktail anesthesia (1.5 mL/kg) with ketamine (100 mg/mL) and xylazine (20 mg/mL). Intravitreal injections were performed with pulled borosilicate glass micropipettes of approximately 40 μm tip diameter (P97 Sutter Instruments, Novato, CA, USA) connected to an electronic pump. Mice were sacrificed 48 h after sodium pentobarbital injection (1.5 mL). The eyes were enucleated and the lenses removed. 

### 2.8. Blood Glucose

Blood glucose levels were checked once a week by Contour XT (Bayer, Leverkusen, Germany) with Contour 25 test strips (Bayer, Leverkusen, Germany), following the manufacturer’s instructions.

### 2.9. Glycated Hemoglobin (HbA1c)

At the end of the experiment, 5 μL of blood samples were used for glycated hemoglobin assessment by using the Mouse Hemoglobin A1c Assay Kit (Crystal Chem, Elk Grove Village, IL, USA). The absorbance was measured at 700 nm using a Victor X5 spectrophotometer (Perkin Elmer, Madrid, Spain).

### 2.10. Protein Isolation and Western Blot 

ARPE-19 cells were collected in a RIPA buffer (ThermoFisher, Waltham, MA, USA) and protease/phosphatase inhibitor cocktail (Sigma-Aldrich, St. Louis, MO, USA). The complete eye, without the lens, was homogenized using the IKA^®^ Ultra-turrax^®^ (Sigma-Aldrich, St. Louis, MO, USA) in a RIPA buffer (ThermoFisher, Waltham, MA, USA) and protease/phosphatase inhibitor cocktail (Sigma-Aldrich, St. Louis, MO, USA). To ensure thorough lysis, the samples were sonicated with three 5 min pulses (5 min ultrasounds–5 min ice). The protein concentration was ascertained using the bicinchoninic acid (BCA) colorimetric assay (ThermoFisher, Waltham, MA, USA). An equal amount of protein (30–40 μg) was loaded onto a 4% stacking–12% resolving SDS-Polyacrylamide gel. The proteins were transferred onto a PVDF membrane (Millipore, Sigma-Aldrich, St. Louis, MO, USA). The membrane was blocked with 3% skimmed milk for 1 h. Overnight incubation with primary antibodies against the VEGFA (1:200 sc-53462 and sc-152, Santa Cruz Biotechnology, Dallas, TX, USA) and β-actin (1:1000, sc-47778, Santa Cruz Biotechnology, Dallas, TX, USA) was used as a loading control. Finally, the membranes were incubated for 1 h with anti-mouse IgG-HRP antibodies (1:10,000, Santa Cruz Biotechnology, Dallas, TX, USA). Visualization was performed with ECL (ThermoFisher, Waltham, MA, USA) and detected with Image Quant LAS-100 mini (GE Healthcare, Chicago, IL, USA). Protein levels were quantified by densitometry using ImageJ (Version 1.54m).

### 2.11. Immunocytochemistry

ARPE-19 cells were seeded on coverslips (TH. Geyer, Hamburg, Germany) in a 24-well plate at a density of 1 × 10^4^ cells per well and incubated for 48 h. Subsequently, the cells were treated as mentioned above. The cells were washed with PBS and fixed with 4% PFA for 5 min. The cells were permeabilized with 0.5% Triton X-100 for 15 min. Afterward, the ARPE-19 cells were blocked with 3% BSA for 1 h and incubated overnight with anti-VEGFA (1:200, sc-7269, Santa Cruz Biotechnology, Dallas, TX, USA) in 3% BSA to prevent nonspecific binding. Cells were washed with PBS 3 times, and the secondary antibody Alexa Fluor 488 goat anti-mouse IgG (1:200, Invitrogen, Darmstadt, Germany) was added and incubated for 1 h at room temperature. Subsequently, ARPE-19 cells were incubated with phalloidin (1:400, Proteintech CoraLite™594, Thermo Fisher Scientific, MA, USA) for 20 min. Finally, for DNA staining, cells were incubated for 10 min with 4,6-diamidino-2-phenylindole (DAPI; Sigma Aldrich, St. Louis, MO, USA). Fluorescence images were recorded with a laser scanning inverted microscope DM IL LED (Leica Microsystems, Wetzlar, Germany), and images were processed with Las X software.

### 2.12. RNA Isolation from ARPE-19 Cells

RNA was isolated from the ARPE-19 cells using an miRNeasy Mini Kit (Qiagen, Germantown, MD, USA) following the manufacturer’s instructions. The total RNA quantity and quality (260/280 absorbance ratio) was assessed using NanoDrop 2000 (Thermo Fisher Scientific, Waltham, MA, USA).

### 2.13. miRNA Expression Analysis

Quantitative real-time PCR (qRT-PCR) was used to analyze the expression profiles of the selected miRNAs. For miRNA expression analysis, 100–300 ng of RNA was retrotranscribed using a TaqMan MicroRNA Reverse Transcription Kit (Applied Biosystems, Foster City, CA, USA) using specific TaqMan RT primers and the thermocycler Verity pro 96-well thermal cycler (Applied Biosystems, Foster City, CA, USA), with cycles of 16 °C/30 min, 42 °C/30 min, 85 °C/5 min, and 4 °C/infinity. The qRT-PCR process was performed using TaqMan™ microRNA Assays (Thermo Fisher Scientific, Waltham, MA, USA) with TaqMan Gene Expression Master Mix (Applied Biosystems, Foster City, CA, USA) and RT-PCR Roche 234 LighterCycler 480 with the appropriate temperature cycles. Normalization was performed using the RNU6B snoRNA. The relative expression was calculated using the 2^−ΔΔCt^ method.

### 2.14. mRNA Expression Analysis

The mRNA expression was analyzed using qRT-PCR. 750 ng of RNA was retrotranscribed with a high-capacity RNA-to cDNA kit (Applied Biosystems, USA) and the thermocycler Mastercycler Nexus gradient (Eppendorf, Hamburg, Germany), with cycles of 65 °C/5 min, 4 °C/5 min, 55 °C/50 min, 85 °C/5 min 37 °C/20 min, and 4 °C/infinity. qRT-PCR was performed using SYBR Green Supermix (Bio-Rad, Hercules, CA, USA), primers (Table 1), and RT-PCR Roche 234 LighterCycler 480 with appropriate temperature cycles. GAPDH was used as an internal reference gene. The relative expression was calculated using the 2^−ΔΔCt^ method.

### 2.15. Vasculogenesis Assay

Pre-cooled 96-well plates were coated with 70 μL Matrigel (Becton Dickinson, Andover, MA, USA). HUVECs were seeded at a density of 3 × 10^4^ cells/cm^2^ and then treated with CCCM for 5 h (see Materials and Methods Section 2.2). 70 μL of the medium was added to each well. Matrigel was allowed to polymerize for 30 min at 37 °C. Images were captured using an Olympus CKX41 inverted microscope (Olympus, Tokyo, Japan) and recorded using an Olympus DP74 digital camera (Olympus, Tokyo, Japan). The total tube length (branch length μm) was automatically quantified by an angiogenesis analyzer plugin from 3 random pictures (1200 × 1800 pixel) for each experimental condition (n = 3) giving 9 samples/condition measured.

### 2.16. Statistical Analysis

The Shapiro–Wilk normality test was used to test whether a variable was normally distributed. Differences between the two experimental groups were analyzed using Student’s *t*-test. The variation between groups was calculated using a one-way analysis of variance (ANOVA) with Tukey’s multiple comparison test. The result of each experiment is presented as mean ± SEM. Statistical significance was set at 0.05. *, **, *** and **** denoted significance at 0.05, 0.01, 0.001, and 0.0001 levels, respectively. Statistical analyses were performed using the GraphPad Prism 9.3.0. software.

## 3. Results 

### 3.1. High Glucose Modifies miR-205-5p and VEGFA Expression Levels in ARPE-19 Cells 

**Figure 1:**
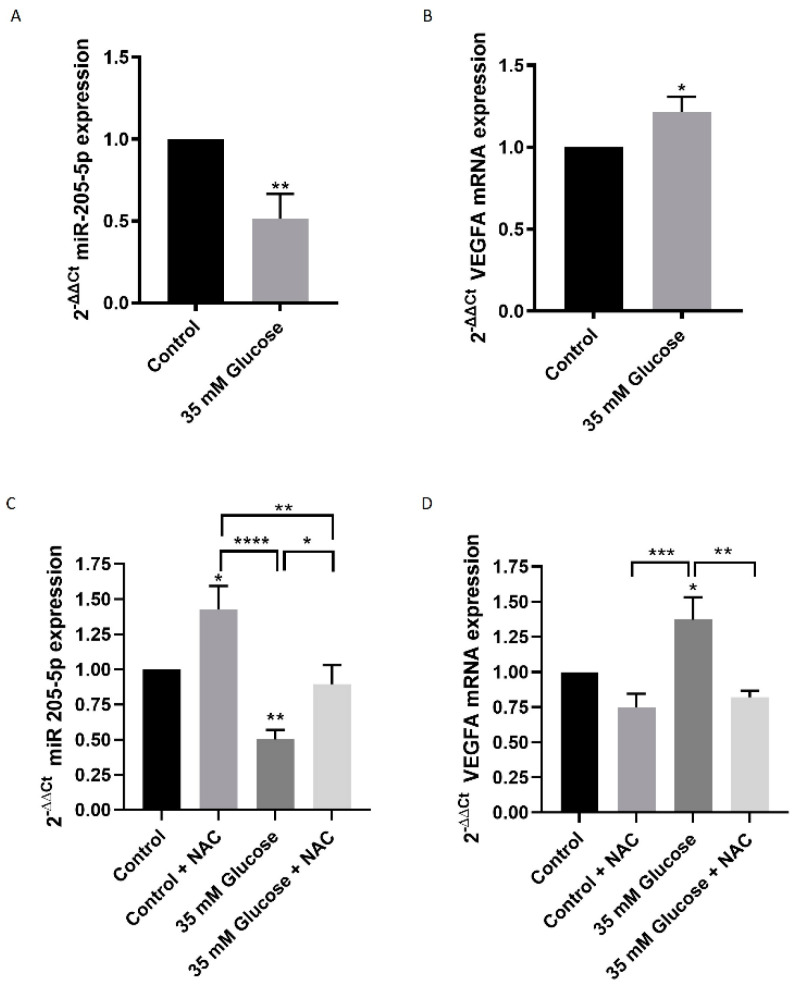
HG-induced miR-205-5p and VEGFA expression levels are restored by NAC in ARPE-19 cells. miR-205-5p (**A**) and VEGFA (**B**) mRNA expression by qRT-PCR from control and 35 mM glucose groups. Values are expressed as mean ± SEM (n = 5). * *p* < 0.05 and ** *p* < 0.01. NAC exposure restored miR-205-5p expression (**C**) and VEGFA mRNA expression in the high glucose group (**D**). Values are expressed as mean ± SEM (n = 5). *p*-value was obtained by ANOVA; * *p* < 0.05, ** *p* < 0.01, *** *p* < 0.001 and **** *p* < 0.0001.

ARPE-19 cells cultured with 35 mM glucose showed a significant reduction in miR-205-5p expression levels compared to the control group (0.324 ± 0.192) (Figure 1A). Correspondingly, the predicted target of miR-205-5p, VEGFA, was significantly upregulated under high glucose conditions compared to the control (1.217 ± 0.091) (Figure 1B). Given that high glucose exposure induces oxidative stress in cells, we investigated whether miR-205-5p expression could be influenced by the antioxidant agent N-acetylcysteine (NAC). Treatment with NAC restored miR-205-5p levels to those observed in the control group (0.895 ± 0.135) (Figure 1C). Furthermore, NAC treatment also led to a significant increase in miR-205-5p levels in the control group (1.426 ± 0.167) (Figure 1C). To further explore the impact of NAC, VEGFA mRNA expression was analyzed after antioxidant exposure. Under high glucose conditions, the NAC treatment significantly reduced VEGFA mRNA expression to levels comparable to the control group (0.821 ± 0.046) (Figure 1D).

### 3.2. miR-205-5p Restores VEGFA Expression Levels in ARPE-19 Cells

**Figure 2:**
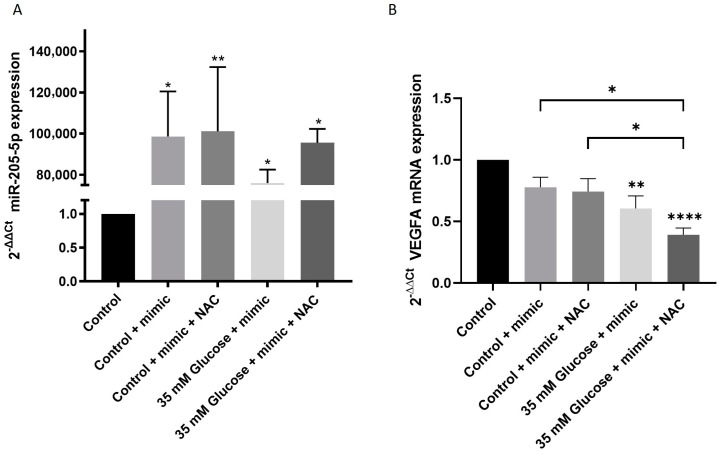
miR-205-5p restores VEGFA mRNA expression levels in ARPE-19 cells. ARPE-19 cells were cultured with control or high glucose media for 5 d. Cells were then transfected with miR-205-5p mimic during 48 h. miR-205-5p (**A**), and VEGFA mRNA (**B**) expression levels were detected by qRT-PCR. Values are expressed as mean ± SEM (n = 4). *p*-value was obtained by one-way ANOVA; * *p* < 0.05, ** *p* < 0.01 and **** *p* < 0.0001.

Hyperglycemia upregulates VEGFA expression levels, leading to a decreased expression of miR-205-5p. To evaluate the role of miR-205-5p as a modulator of VEGFA mRNA, ARPE-19 cells were transfected with a miR-205-5p mimic after 5 days of high glucose exposure. Transfection with the miR-205-5p mimic led to a significant increase in miR-205-5p expression across all conditions (Figure 2A). This intervention restored VEGFA mRNA expression to levels comparable to the control group (Figure 2B). No significant differences were observed between the NAC + miR-205-5p mimic group and other treatments (NAC alone or miR-205-5p mimic alone).

**Figure 3:**
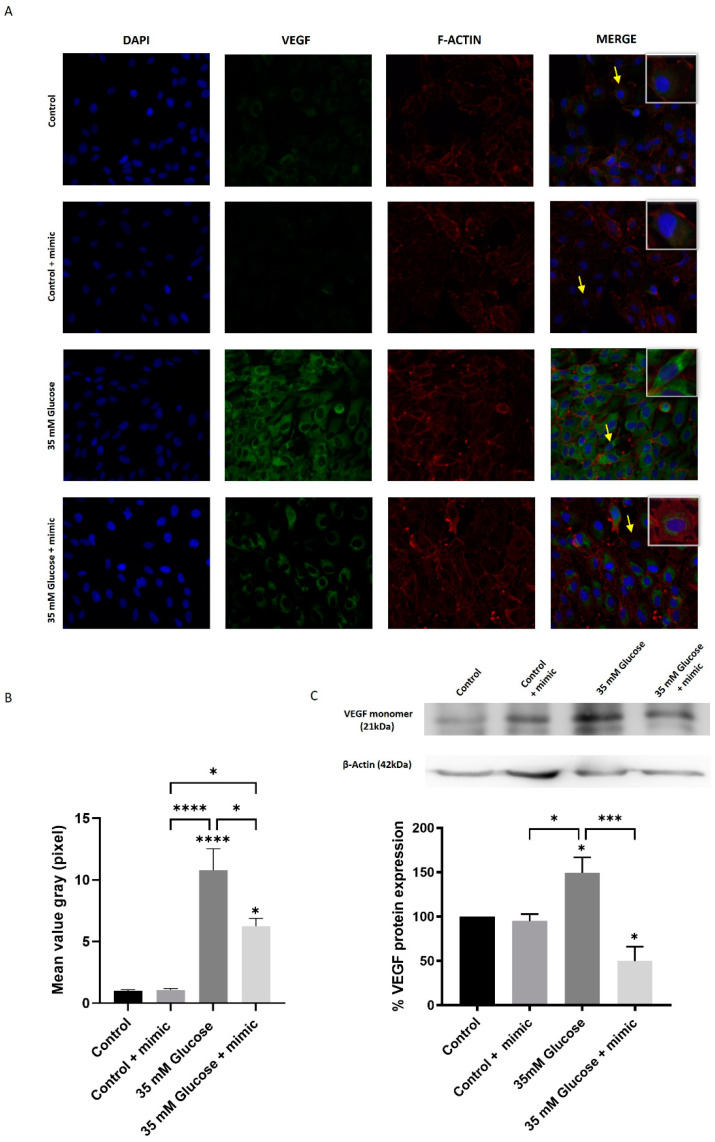
miR-205-5p restores VEGFA protein expression in ARPE-19 cells. ARPE-19 cells were cultured with control or high glucose media for 5 d. Cells were then transfected with miR-205-5p mimic during 48 h. Then, cells were conducted for immunocytochemistry against VEGFA (green), F-Actin (Red) DAPI (blue) (**A**). Immunofluorescence was assessed by Las X software (**B**). Western blot analysis of VEGFA protein was performed after transfection with miR-205-5p mimetic (**C**). Values are expressed as mean ± SEM (n = 4). *p*-value was obtained by one-way ANOVA; * *p* < 0.05, *** *p* < 0.001 and **** *p* < 0.0001.

To determine whether changes in VEGFA mRNA expression translated to protein expression, Western blot and immunocytochemistry analyses were conducted in ARPE-19 cells. Immunocytochemistry revealed weak cytoplasmic immunofluorescence for VEGFA-positive cells in both the control and control + mimic groups (Figure 3A,B). In contrast, cells exposed to 35 mM glucose exhibited strong cytoplasmic labeling. The miR-205-5p mimic administration significantly reduced VEGFA-positive labeling compared to the glucose-treated group (Figure 3A,B). These findings were corroborated by the Western blot analysis, which confirmed a reduction in VEGFA protein levels following the miR-205-5p mimic transfection (Figure 3C).

### 3.3. miR-205-5p Regulates High Glucose-Induced Angiogenesis in HUVEC Cells

**Figure 4:**
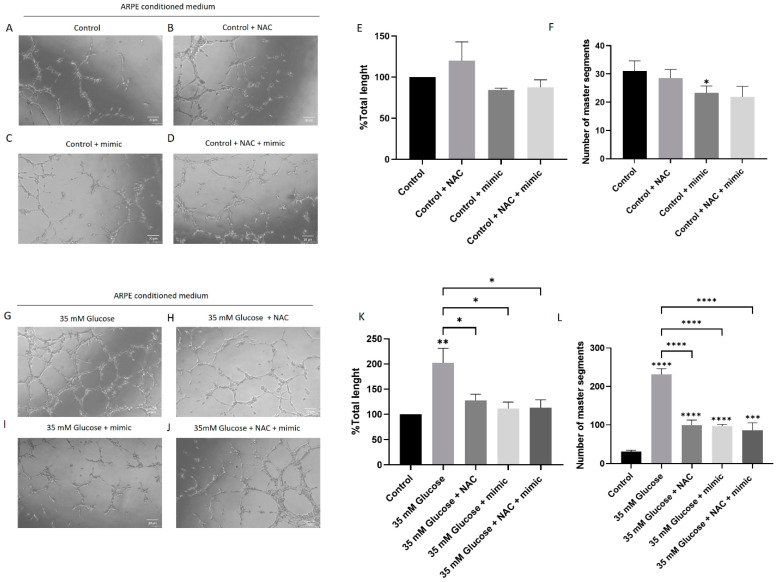
miR-205-5p regulates High Glucose-induced tube formation in HUVEC cells. HUVEC cell tube formation under different cell culture-conditioned media (CCCM) obtained from ARPE-19 cell cultures: control medium (**A**), control +NAC (**B**), control + miR-205-5p mimic (**C**), control +NAC+ miR-205-5p mimic (**D**), 35 mM glucose treated (**G**), 35 mM glucose medium +NAC (**H**), 35 mM glucose + miR-205-5p mimic (**I**), and 35 mM glucose + NAC + miR-205-5p mimic (**J**). Total length (**E**,**K**) and number of master segments (**F**,**L**) were obtained from all experimental conditions (n = 3) (3 × 3 = 9 samples/experimental condition) by using ImageJ 1.54g software. Values are expressed as mean ± SEM (n = 3). *p*-value was obtained by one-way ANOVA; * *p* < 0.05, ** *p* < 0.01, *** *p* < 0.001 and **** *p* < 0.0001.

High glucose conditions promote angiogenesis by enhancing cell migration and proliferation. To investigate the role of miR-205-5p in angiogenesis, XTT, Matrigel, and scratch assays were performed using an in vitro model. HUVECs were treated with cell culture-conditioned media (CCCM) as described in the Materials and Methods Section 2.2. Tube formation in HUVECs, quantified by measuring total length and master segments, was assessed as an indicator of angiogenesis. Minimal tubular processes were observed under control conditions, with a further reduction in master segments in the control + mimic group compared to the control alone (Figure 4A–F). In contrast, the High glucose -conditioned medium significantly increased tube formation (202.4 ± 28.7) (Figure 4G–L). This increase was normalized by an NAC-conditioned medium (127.9 ± 12.24), an miR-205-5p mimic-conditioned medium (111.7 ± 12.57), and an NAC + miR-205-5p mimic-conditioned medium (113.1 ± 15.53) (Figure 4K–L).

**Figure 5:**
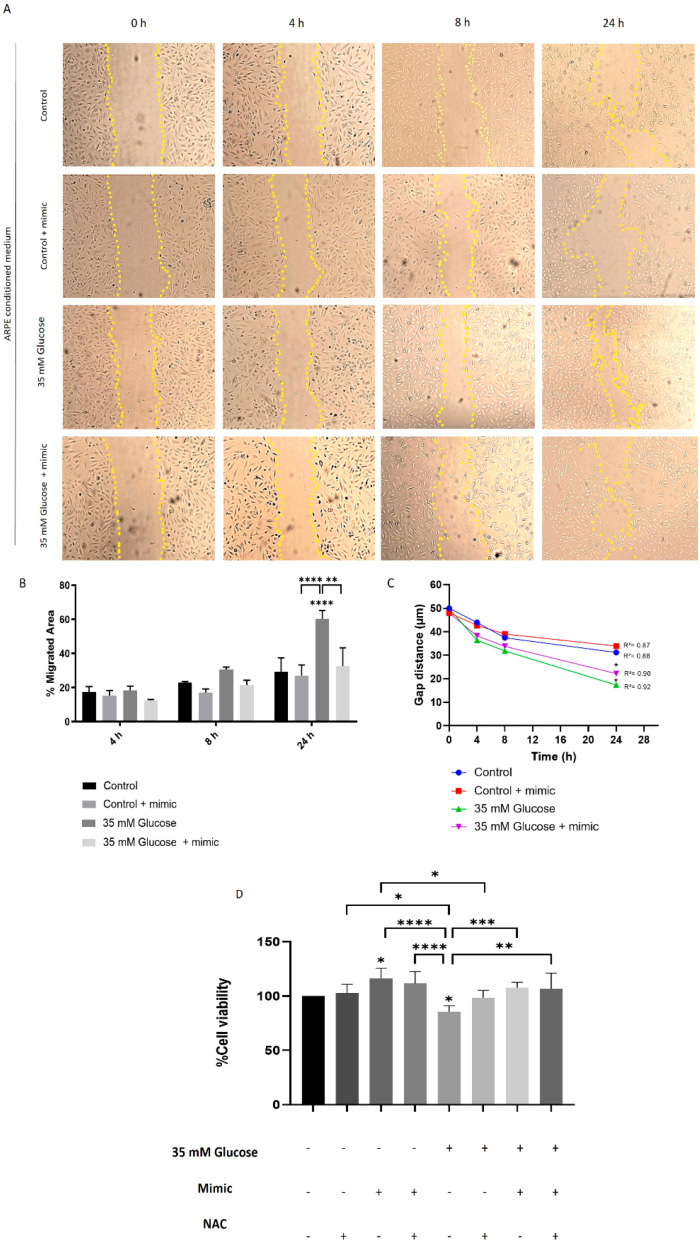
HUVEC cells’ migration is hindered by miR-205-5p. Representative images were captured at 0, 4, 8, and 24 h after HUVEC migration under various conditions (**A**). Quantification of cell migration (**B**,**C**). ARPE-19 cell viability was assessed using the XTT assay (**D**). Values are presented as mean ± SEM (Migration n = 4 and viability n = 6). *p*-value was determined using one-way ANOVA; * *p* < 0.05, ** *p* < 0.01, *** *p* < 0.001 and **** *p* < 0.0001.

The High glucose-conditioned medium also significantly enhanced HUVEC migration compared to control conditions (Figure 5A,B). Treatment with NAC- or miR-205-5p-conditioned media restored HUVEC migration to control levels, consistent with the Matrigel results (Figure 5A–C). Additionally, cell viability, which was significantly reduced under High glucose conditions, was restored by NAC, miR-205-5p mimic, or their combination (Figure 5D).

### 3.4. Alloxan-Induced Diabetic Mice Model

**Figure 6:**
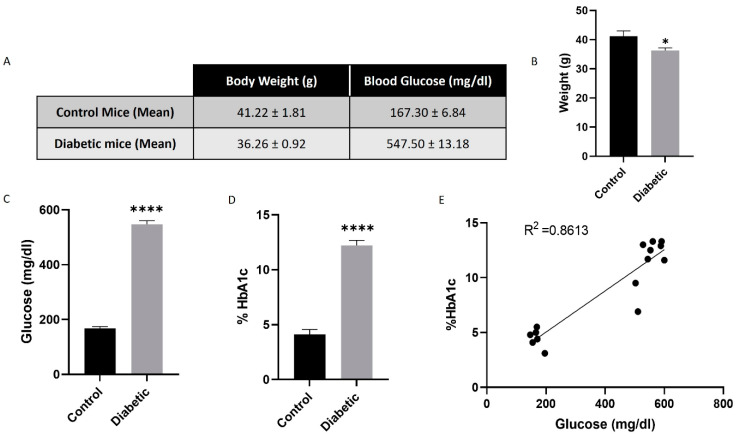
Alloxan-induced diabetic mice model. Alloxan significantly decreased weight 4 weeks after injection (n = 5) (**A**,**B**). Alloxan significantly increased high glucose circulating levels in mice compared to control animals (n = 6) (**C**). Glycated hemoglobin (HbA1c) (as a percentage) confirmed hyperglycemic conditions in mice compared to control (n = 7) (**D**). Spearman’s correlation between high glucose circulating levels and glycated hemoglobin (n = 15) (**E**). Values are presented as mean ± SEM. *p*-value was determined using *t*-test; * *p* < 0.05 and **** *p* < 0.0001.

A significant weight loss was observed in diabetic animals compared to the control group (36.26 ± 0.92) (Figure 6A,B). Alloxan administration led to elevated blood glucose levels (>500 mg/dL) within 24 h post-injection, significantly higher than those in the control group (547.5 ± 13.18) (Figure 6A,C). Four weeks after diabetes induction, glycated hemoglobin levels were markedly increased in diabetic animals (12.22 ± 0.46) (Figure 6D). Additionally, hyperglycemia levels demonstrated a positive correlation with glycated hemoglobin levels (Figure 6E).

### 3.5. Intravitreal miR-205-5p Mimic Restores VEGFA Levels in Diabetic Mice Model

**Figure 7:**
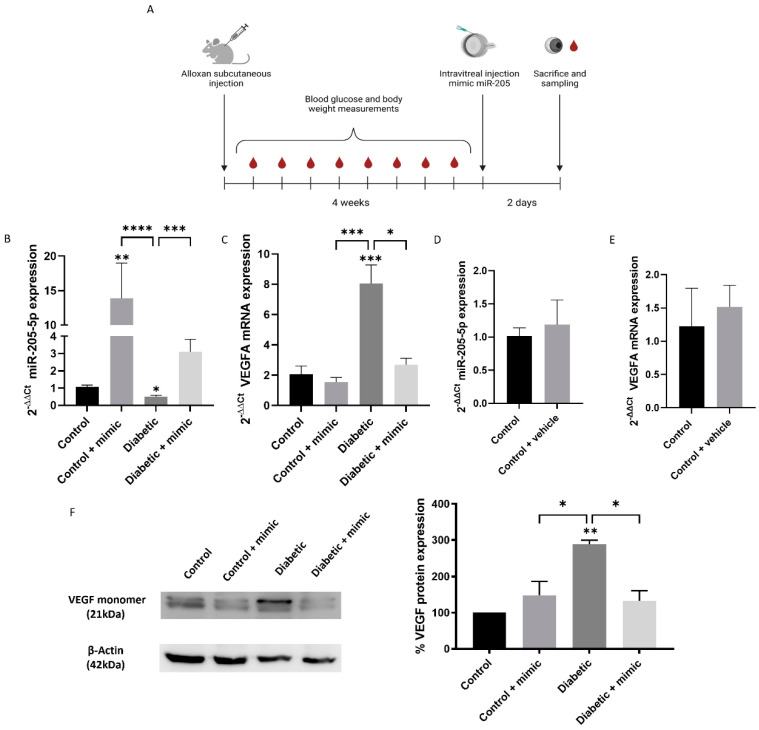
Intravitreal miR-205-5p mimic restores VEGFA mRNA levels. Timeline representation for experimental proceeding (**A**). miR-205-5p levels were significantly decreased in diabetic mice compared to control. Ectopic miR205-5p mimic increased miR-205-5p levels to control ones (**B**) and VEGFA mRNA (**C**) expression levels were increased in diabetic mice and restored by ectopic miR-205-5p mimic administration (n = 4). No significant changes were observed by intravitreal vehicle injection (**D**,**E**) (n = 3). Western blot analysis for VEGFA shows higher levels in diabetic mouse eye (n = 3) (**F**). Values are expressed as mean ± SEM. *p*-value was obtained by one-way ANOVA; * *p* < 0.05, ** *p* < 0.01, *** *p* < 0.001 and **** *p* < 0.0001.

Four weeks after alloxan injection (Figure 7A), eyes from diabetic mice showed a significant reduction in miR-205-5p levels (0.50 ± 0.78) (Figure 7B), while VEGFA mRNA levels were markedly increased compared to the control group (8.06 ± 1.22) (Figure 7C). Intravitreal injection of the miR-205-5p mimic resulted in a three-fold increase in miR-205-5p levels in control mice compared to the vehicle group (13.91 ± 5.08). In diabetic mice, reduced miR-205-5p levels were restored to control values following a mimic injection (3.10 ± 0.73) (Figure 7B). Similarly, elevated VEGFA mRNA levels in diabetic mice were significantly reduced to control levels after mimic administration (2.69 ± 0.43) (Figure 7C). Intravitreal vehicle injections did not significantly alter miR-205-5p or VEGFA mRNA expression compared to naïve animals (Figure 7D,E).

VEGFA mRNA upregulation corresponded to increased VEGFA protein expression, as confirmed by the Western blot analysis. VEGFA protein levels, which were significantly elevated in diabetic mice compared to controls, were normalized after the miR-205-5p mimic injection, consistent with VEGFA mRNA expression patterns (Figure 7F).

## 4. Discussion

Previous studies have highlighted that miR-205-5p is downregulated under oxidative stress conditions, leading to an increase in VEGFA expression [19]. This observation is consistent across different oxidative challenges, including H_2_O_2_ [12] and hyperglycemia [20]. In our study, we observed that high glucose significantly reduced miR-205-5p expression in ARPE-19 cells, mirroring findings from oxidative stress models. Importantly, this downregulation was reversed by treatment with N-acetylcysteine (NAC), a known antioxidant that replenishes glutathione levels, restoring miR-205-5p expression to control levels. These results suggest that high glucose and oxidative stress converge to suppress miR-205-5p expression, likely via mechanisms linked to redox imbalance [21].

The relationship between miR-205-5p and VEGFA was further confirmed in our experiments. In line with previous studies, high glucose conditions led to an upregulation of VEGFA. Our data revealed that restoring miR-205-5p expression via a mimic transfection significantly normalized VEGFA mRNA and protein levels, indicating a direct regulatory link between miR-205-5p and VEGFA under hyperglycemic conditions. Additionally, miR-205-5p has been shown to inhibit angiogenesis and migration, highlighting its potential role in counteracting pathological neovascularization and cellular motility associated with elevated VEGFA levels.

One potential mechanism underlying the downregulation of miR-205-5p under hyperglycemic conditions involves the interplay of oxidative stress [19], epigenetic modifications [22], and transcriptional regulation [17]. Prolonged hyperglycemia is known to induce oxidative stress and hypoxia, which can significantly alter DNA methylation patterns and chromatin structure [23]. These epigenetic changes may disrupt the transcription of the MIR205 gene [24], particularly through the involvement of transcription factors such as HIF-1α and Sp1 [17], both of which are activated under hyperglycemic stress [25]. HIF-1α is particularly noteworthy as it directly upregulates VEGFA expression, linking hypoxia and angiogenesis pathways, which are central to the pathogenesis of diabetic retinopathy (DR) [26]. Additionally, long non-coding RNAs (lncRNAs) such as MALAT1 may act as molecular sponges, sequestering miR-205-5p and further reducing its functional availability. A recent study conducted in retinal endothelial cells demonstrated that MALAT1 sponges miR-205-5p, exacerbating VEGFA overexpression and contributing to angiogenesis under hyperglycemic conditions [19]. Furthermore, lncRNAs have been implicated in suppressing miR-205-5p availability, thereby exacerbating hyperglycemia-induced proliferation and inflammation [27].

The interplay between insulin signaling, miR-205-5p, and VEGFA highlights a critical regulatory network in angiogenesis under hyperglycemic conditions. miR-205-5p modulates insulin sensitivity by targeting FOXO1 [28], and its downregulation under hyperglycemia disrupts normal insulin signaling, promoting metabolic dysregulation and increased VEGFA expression. VEGFA, a key driver of angiogenesis, is further upregulated under oxidative stress and hypoxia, contributing to vascular permeability and neovascularization in DR. Additionally, studies in diabetic islets reveal that miR-205-5p overexpression impairs insulin secretion by targeting Tcf7l2, suggesting its dual role in modulating both metabolic and angiogenic pathways [29].

Several studies have reported reduced expression levels of miRNAs associated with DR in plasma, which may be implicated in the progression and severity of this diabetes-related complication [30]. This systemic downregulation of miRNAs, including miR-205-5p, could potentially influence their expression in ocular tissues, contributing to the pathological processes underlying DR. Furthermore, miR-205-5p has been specifically found to be downregulated in extracellular vesicles in the context of diabetes [15,18]. This systemic reduction highlights the potential role of miR-205-5p in endocrine regulation, suggesting that its diminished extracellular availability may exacerbate metabolic and vascular dysregulation commonly observed in diabetic complications [18].

The microRNA miR-205-5p is essential for maintaining the integrity of epithelial cells [31], including retinal pigment epithelium (RPE) cells. Its downregulation can compromise the epithelial characteristics of these cells, facilitating the epithelial-to-mesenchymal transition (EMT) [32], particularly under hyperglycemic conditions [19]. Studies have shown that in ARPE-19 cells exposed to high glucose concentrations, the reduction of miR-205-5p promotes migratory processes and tube formation [15] indicative of EMT. Moreover, the overexpression of miR-205-5p reverses these effects, highlighting its protective role against hyperglycemia-induced EMT. Similarly, research in keratinocytes has demonstrated that the overexpression of miR-205-5p increases E-cadherin expression and reduces mesenchymal markers such as N-cadherin and α-SMA, indicating a reversal of EMT [27]. 

The miR-205-5p exhibits a significant anti-angiogenic effect by targeting VEGFA, as demonstrated in several types of cancers. For instance, in breast cancer, the overexpression of miR-205-5p reduces cell proliferation, migration, and endothelial tube formation by negatively regulating the Wnt/β-catenin pathway [33]. In hepatocellular carcinoma [34], gastric cancer [13], and bladder cancer [35], miR-205-5p has been identified as a key regulator of angiogenesis through VEGFA modulation. Interestingly, miR-205-5p is overexpressed in certain conditions where it promotes disease progression. In cutaneous squamous cell carcinoma [36], esophageal squamous cell carcinoma [37], and neck squamous cell carcinoma [38], these findings suggest that the role of miR-205-5p is highly context-dependent, varying across tissues and disease states.

However, a significant limitation in the field of ophthalmology is that most of these studies have been conducted in the context of cancer biology, leaving a gap in understanding how miR-205-5p functions in ocular diseases such as DR or age-related macular degeneration [19]. Although VEGFA serves as a central mediator in both cancer and ocular neovascular diseases, the unique microenvironment of the retina, coupled with the specialized functions of RPE cells, introduces specific complexities [2].

According to previous studies and the findings from our work, miRNAs such as miR-203a-3p, miR-126, and miR-205-5p have demonstrated potent anti-angiogenic effects, largely through their ability to directly bind to the 3′UTR of VEGFA mRNA and downregulate its expression. For example, Han et al. showed that the intravitreal administration of miR-203a-3p in an oxygen-induced retinopathy model significantly reduced VEGFA and HIF-1α expression, mitigating retinal neovascularization [39]. Similarly, Fan et al. reported that miR-126 inhibited pathological retinal angiogenesis in a retinopathy of prematurity in an in vivo model, demonstrating its potential to suppress VEGFA-driven neovascular growth [40].

In line with these findings, our study shows that miR-205-5p, when administered intravitreally as a mimic, restores VEGFA levels and reduces angiogenic processes both in vitro and in vivo, as demonstrated in the diabetic mouse model. While these miRNAs share overlapping mechanisms in VEGFA regulation, miR-205-5p offers an additional layer of regulation by its interplay with oxidative stress pathways, further enhancing its potential therapeutic relevance.

However, while miRNAs show great promise, limitations persist in their translation to clinical settings, particularly in ophthalmology. Intravitreal delivery methods, though effective, pose risks such as inflammation and endophthalmitis, and their repeated use can diminish patient compliance [41]. Future studies should explore alternative delivery strategies to improve patient safety and evaluate the long-term outcomes of miRNA therapies in preclinical models, as these advancements are critical for their integration into routine clinical practice.

## 5. Conclusions

In summary, our findings underscore the therapeutic potential of miR-205-5p in mitigating hyperglycemia-induced VEGFA overexpression and pathological angiogenesis. Through intravitreal administration and in vitro mimic transfection, miR-205-5p effectively restored VEGFA levels and inhibited key processes such as angiogenesis and migration, which are central to the progression of diabetic retinopathy. These results highlight miR-205-5p as a promising candidate for developing RNA-based therapies targeting VEGFA, particularly for patients unresponsive to current antibody-based treatments.

## Figures and Tables

**Table 1:** mRNA primers for qRT-PCR.

mRNA	Forward	Reverse
VEGFA	5′-TGAAGGTCGGAGTCAACGGAT-3′	5′-TTCTCAGCCTTGACGGTGCCA-3′
GAPDH	5′-GACTTATACCGGGATTTCTTG-3′	5′-AATGTGAATGCAGACCAAAG-3′

## Data Availability

Data can be provided upon appropriate request.
